# Supplementary material for: Enhanced Levels of Peroxisome-Derived H2O2 Do Not Induce Pexophagy but Impair Autophagic Flux in HEK-293 and HeLa Cells
Source: Antioxidants (Basel). 2023 Mar 2;12(3):613. doi: 10.3390/antiox12030613 (PMC10045779; doi:10.3390/antiox12030613)
Supplement: Supplementary file 1 [file antioxidants-12-00613-s001.zip › antioxidants-2229989-supplementary.pdf]

antioxidants

MDPI

Supplementary Materials

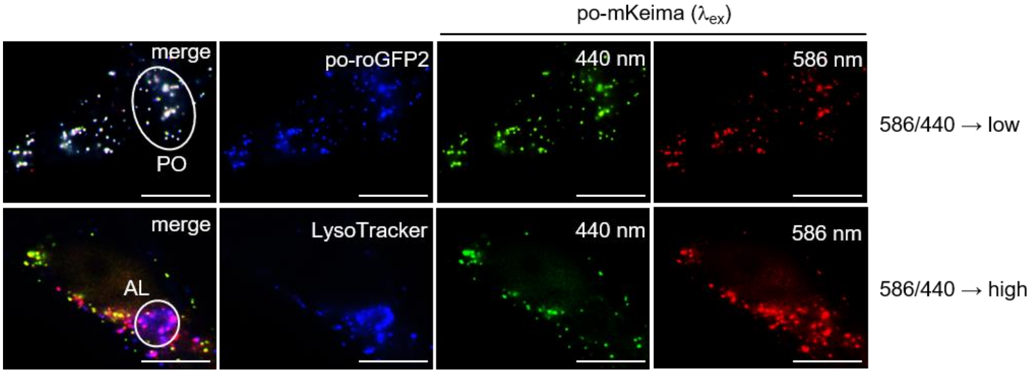

Figure S1. Validation of po-mKeima in living DD-DAO Flp-In T-REx HeLa cells. Colocalization analysis between the peroxisomal marker po-roGFP2 (false color: blue) or the acidotropic fluorescent probe LysoTracker (false color: blue) and po-mKeima excited at 440 nm (false color: green) or 586 nm (false color: red). The white-yellowish (= low 586/440 excitation peak ratio) and reddish (= high 586/440 excitation peak ratio) dots represent peroxisomes (PO) and autolysosomes (AL), respectively. Scale bar, 10 µm.

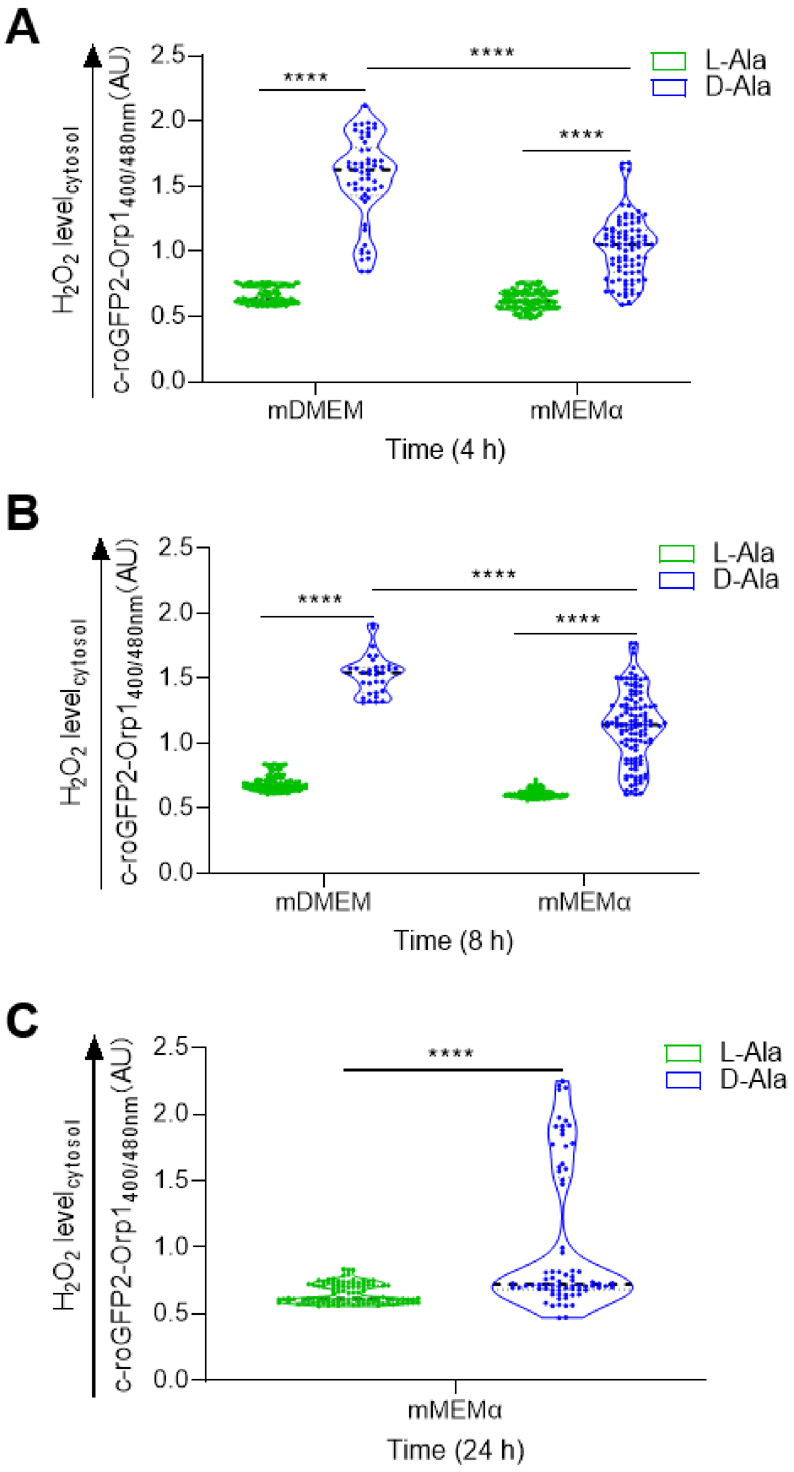

Figure S2. Effect of assay medium on peroxisomal H_2O_2 release in HEK-293 cells. DD-DAO Flp-In T-REx 293 cells were processed as described in the legend to Figure 3. The response ratios of c-roGFP2-Orp1 were monitored at (**A**) 4 h, (**B**) 8 h, and (**C**) 24 h. Note that only those conditions were quantified in which the cells show an overall healthy appearance. The data obtained for the D-Ala conditions were statistically compared as indicated in the graph (****, $p < 0.0001$).

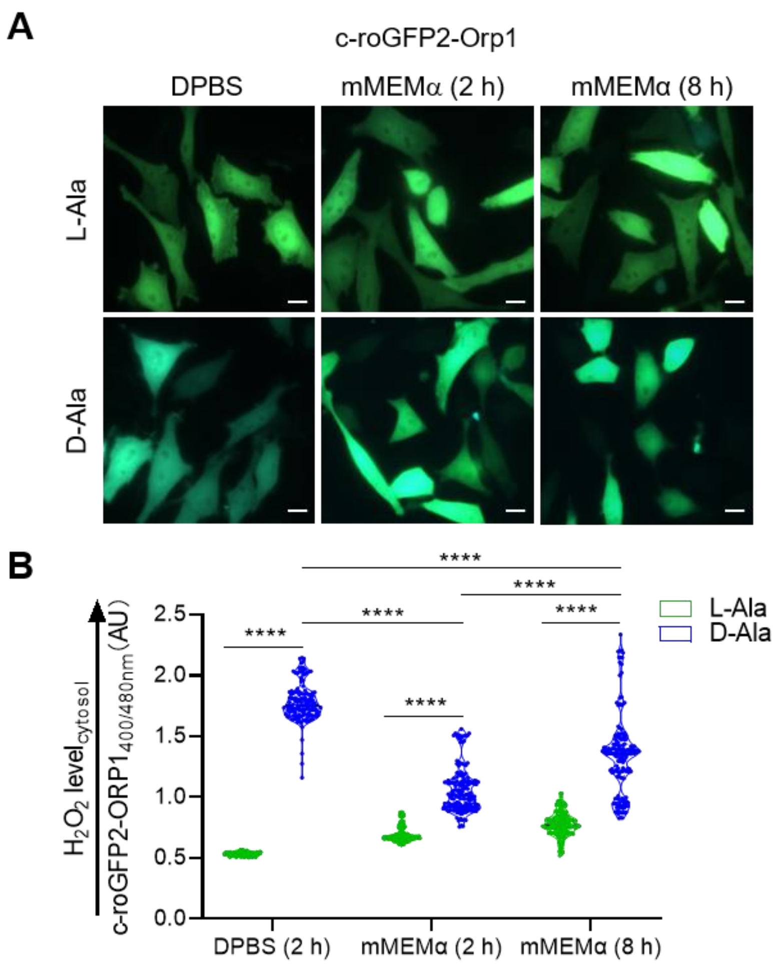

Figure S3. Effect of assay medium on peroxisomal H_2O_2 release in HeLa cells. DD-DAO Flp-In T-REx HeLa cells were cultured and transfected as described in the legend to Figure 3. Next, the cells were incubated in DPBS or mMEM*α* (for media details, see Materials and Methods) and, after the addition of L- or D-Ala, the cytosolic H_2O_2 levels were monitored at the indicated times. (**A**) Representative fluorescence overlay images of c-roGFP2-ORP1 upon excitation at 400 (false color: blue) and 480 (false color: green) nm. Scale bar, 10 µm. (**B**) Response ratios of c-roGFP2-Orp1. The data obtained for the D-Ala conditions were statistically compared as indicated in the graph (****, $p < 0.0001$).

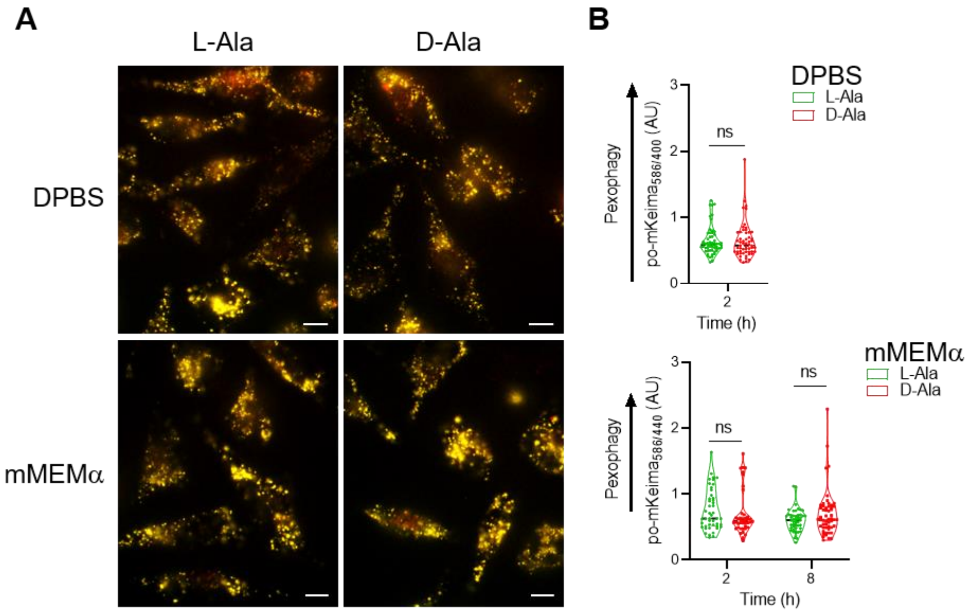

Figure S4. Peroxisome-derived H_2O_2 does not trigger pexophagy in po-mKeima/DD-DAO Flp-In T-REx HeLa cells. DD-DAO Flp-In T-REx HeLa cells stably expressing po-mKeima were cultured as described in the legend to Figure 3. The cells were subsequently transferred to different DPBS or mMEMα (for media details, see Materials and Methods) supplemented with 10 mM L- or D-Ala. (**A**) Representative fluorescence overlay images of po-mKeima upon excitation at 440 (false color: green) and 586 (false color: red) nm. Scale bar, 10 µm. (**B**) 586/440 nm excitation fluorescence ratios of po-mKeima. Data represent the values of 3 independent biological replicates. The corresponding D- and L-Ala data were statistically compared, but no significant differences were found (ns, non-significant).

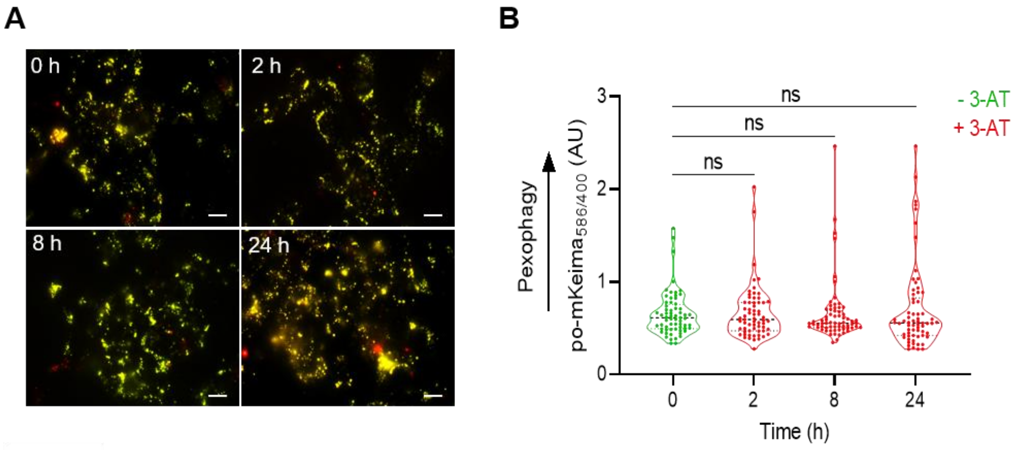

Figure S5. Culturing HEK-293 cells in the presence of 3-amino-1,2,4-triazole does not induce pexophagy. DD-DAO Flp-In T-REx 293 cells stably expressing po-mKeima were cultured in rMEMα medium supplemented with 10 mM 3-amino-1,2,4-triazole (3-AT), and the cells were imaged at the indicated time points. (**A**) Representative fluorescence overlay images of po-mKeima upon excitation at 440 (false color: green) and 586 (false color: red) nm. Scale bar, 10 µm. (**B**) 586/440 nm excitation fluorescence ratios of po-mKeima. Data represent the values of at least 40 randomly selected cells. All conditions were statistically compared to time 0 h, but no significant differences were found (ns, non-significant).

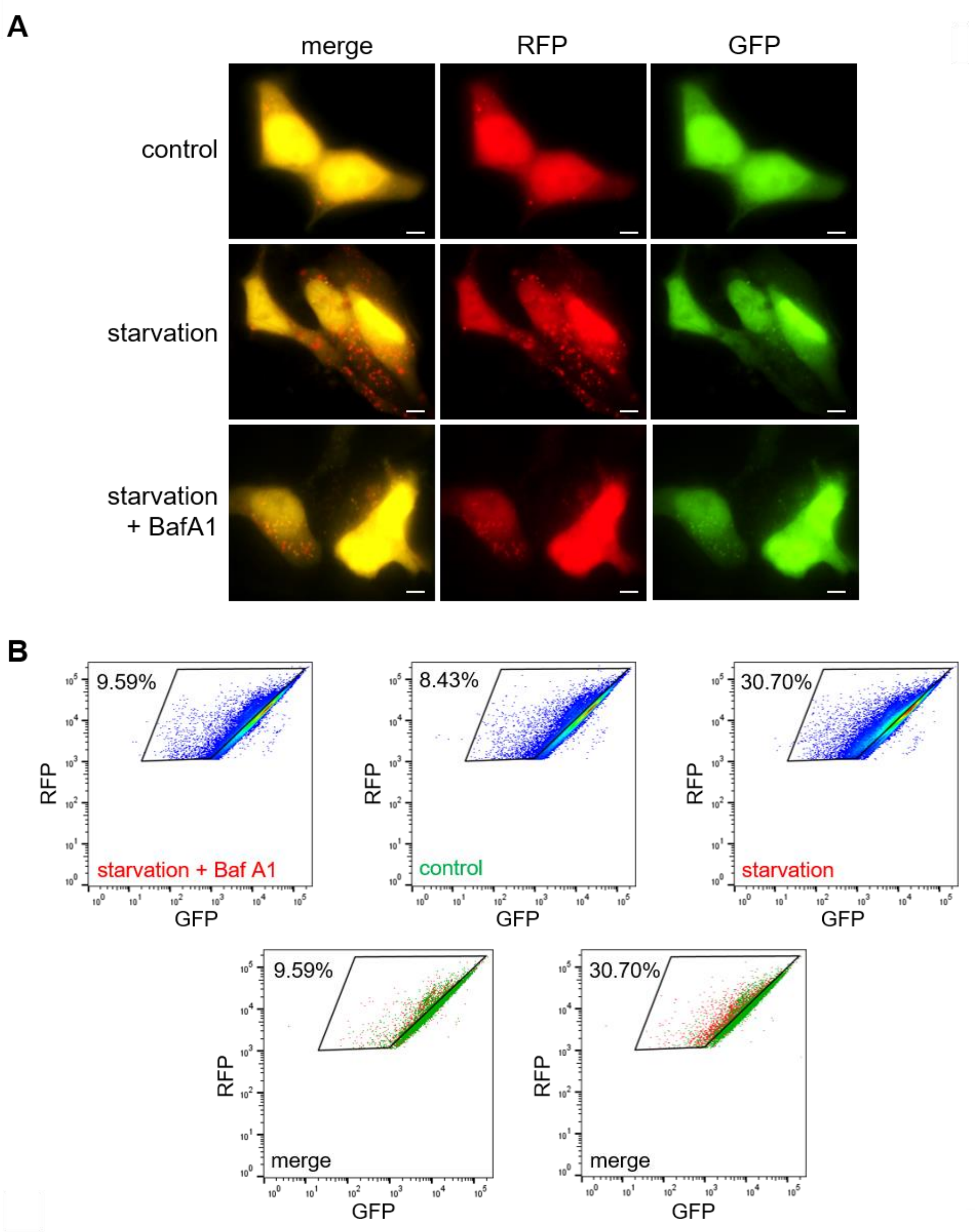

Figure S6. Validation of the autophagic flux reporter mRFP-GFP-LC3 in HEK-293 cells. DD-DAO Flp-In T-REx 293 cells were transfected with a plasmid encoding mRFP-GFP-LC3 and seeded into (Fluoro)dishes containing rMEMα. One day later, the medium was replaced with fresh rMEMα (control) or EBSS (starvation), supplemented or not with 200 nM BafA1. After 8 h, the cells were processed for fluorescence microscopy or FACS analysis. (**A**) Representative images documenting the subcellular distribution patterns of mRFP-GFP-LC3. Scale bar, 10 µm. (**B**) Representative flow cytometry plots. To measure the percentage of cells undergoing autophagy, mRFP-GFP-LC3-positive single-cell populations were gated for a decrease in GFP fluorescence.

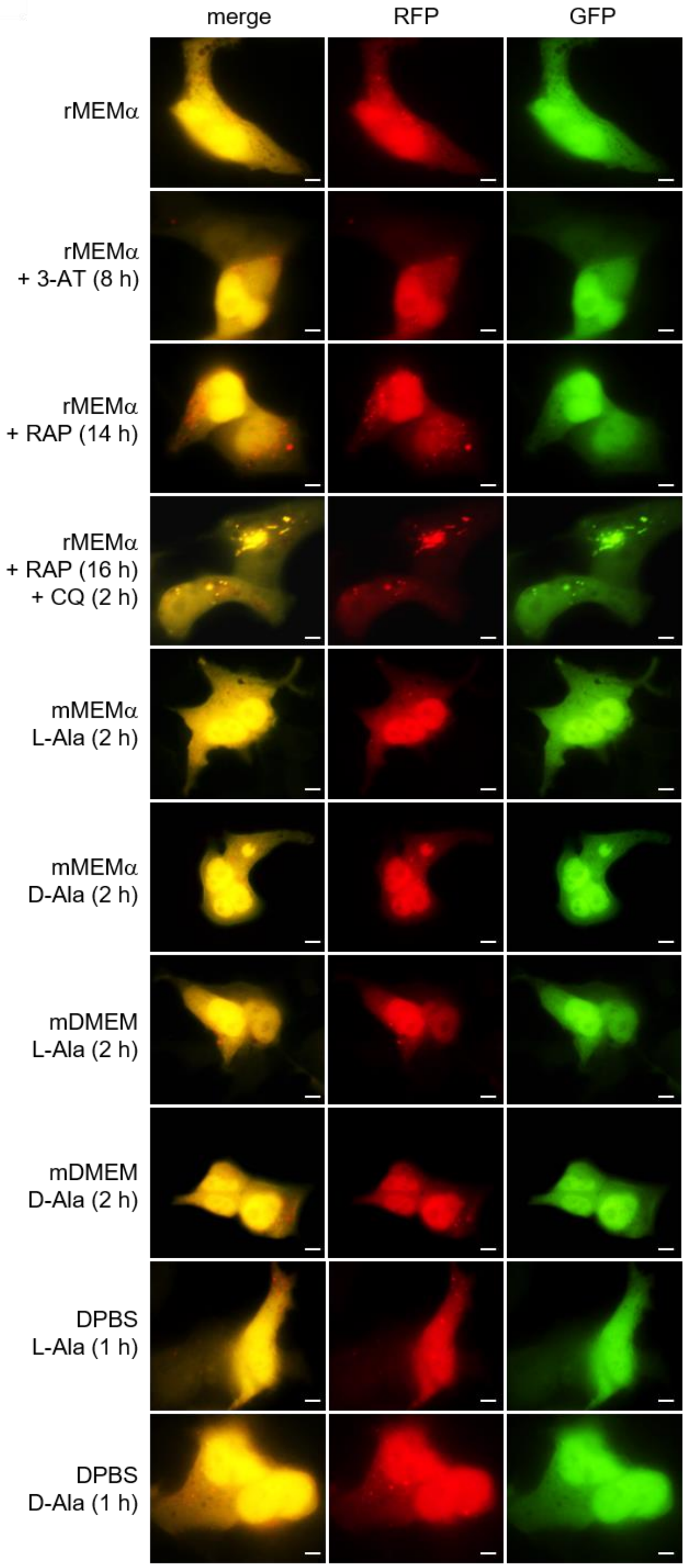

Figure S7. Representative images documenting the subcellular distribution patterns of mRFP-GFP-LC3B in DD-DAO Flp-In T-REx 293 cells assayed under the indicated conditions. For media details, see Materials and Methods. To produce po-H_2O_2, the cells were first cultured as described in the legend to Figure 3. 3-AT, 10 mM 3-amino-1,2,4-triazole; CQ, 200 µM chloroquine; D-Ala, 20 mM D-alanine; L-Ala, 20 mM L-alanine; RAP, 50 nM rapamycin. Scale bar, 10 µm.

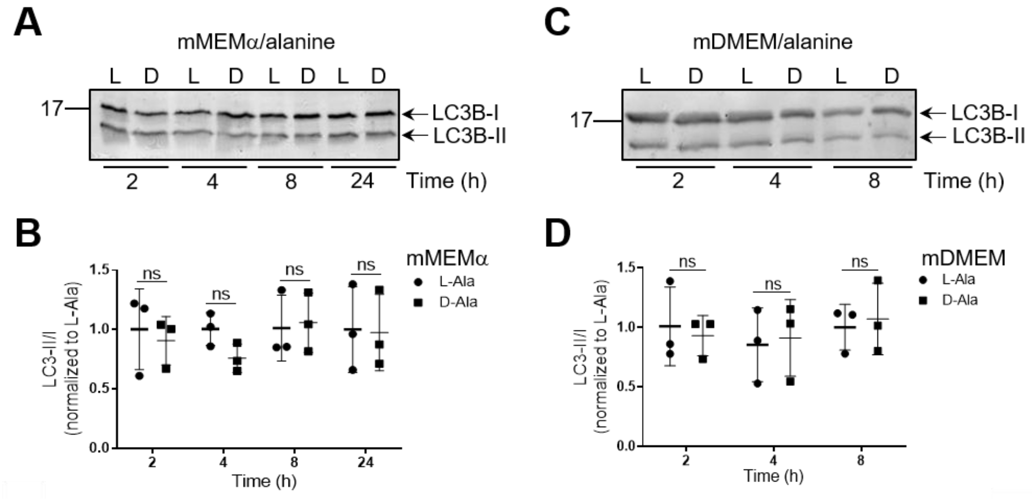

Figure S8. Moderate levels of po-H_2O_2 do not affect the processing of LC3B-I to LC3B-II. DD-DAO Flp-In T-REx 293 cells were cultured as described in the legend to Figure 3. Next, the cells were transferred to mMEMα or mDMEM (for details, see Materials and Methods) supplemented with 10 mM L- or D-Ala. At the indicated time points, the cells were processed for immunoblotting with an antibody specific to LC3B. (**A**,**C**) Representative immunoblots. (**B**,**D**) Quantifications of the LC3B-II/LC3B-I ratios. The corresponding D- and L-Ala data were statistically compared (ns, non-significant).

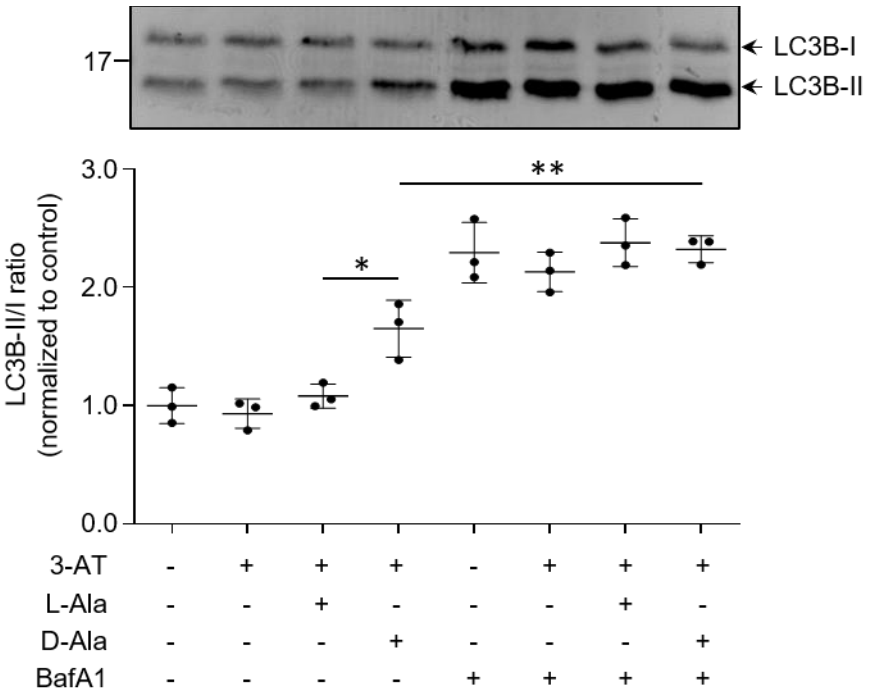

Figure S9. Severe po-H_2O_2 insults cause a late-stage impairment of autophagy. DD-DAO Flp-In T-REx 293 cells were cultured as described in the legend to Figure 7. A representative immunoblot (upper panels) and quantifications (lower panels) are shown. Data represent means ± SD (n = 3 independent biological replicates). Data sets that were statistically compared are indicated (*, $p < 0.05$; **, $p < 0.01$).

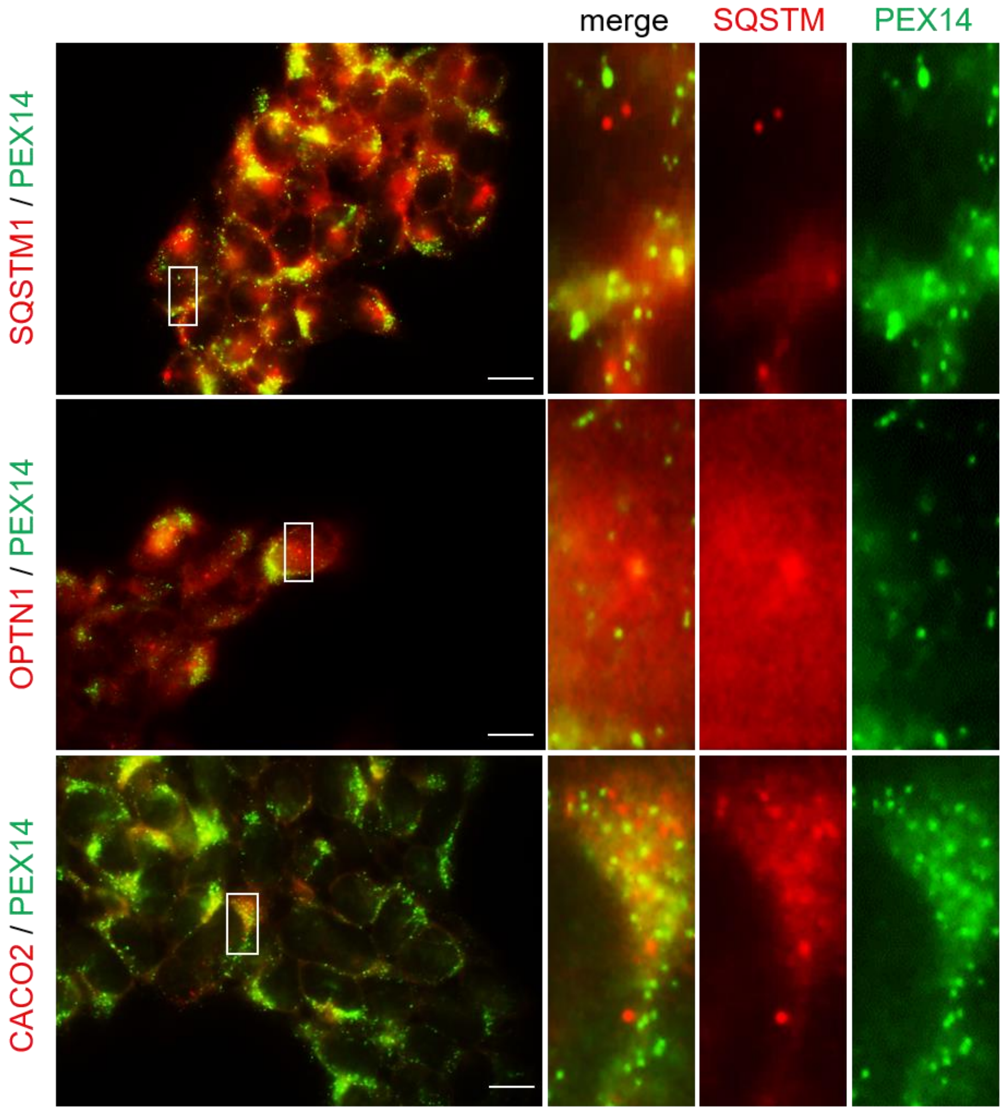

Figure S10. The specific autophagy receptors SQSTM1, OPTN, and CACO2 are not recruited to peroxisomes upon moderate production of po-H_2O_2. DD-DAO Flp-In T-REx 293 cells were cultured as described in the legend to Figure 3. Next, the cells were incubated in mDMEM containing 20 mM L- or D-Ala. After 2 h, the cells were fixed and processed for immunofluorescence microscopy using a mouse antiserum against PEX14 and rabbit antisera against SQSTM1, OPTN, or CACO2 in combination with goat anti-mouse and anti-rabbit secondary antibodies conjugated to Alexa Fluor 488 and Texas Red, respectively. The panels shown to the right of each image are magnified views of the boxed areas. Scale bars, 10 µm.

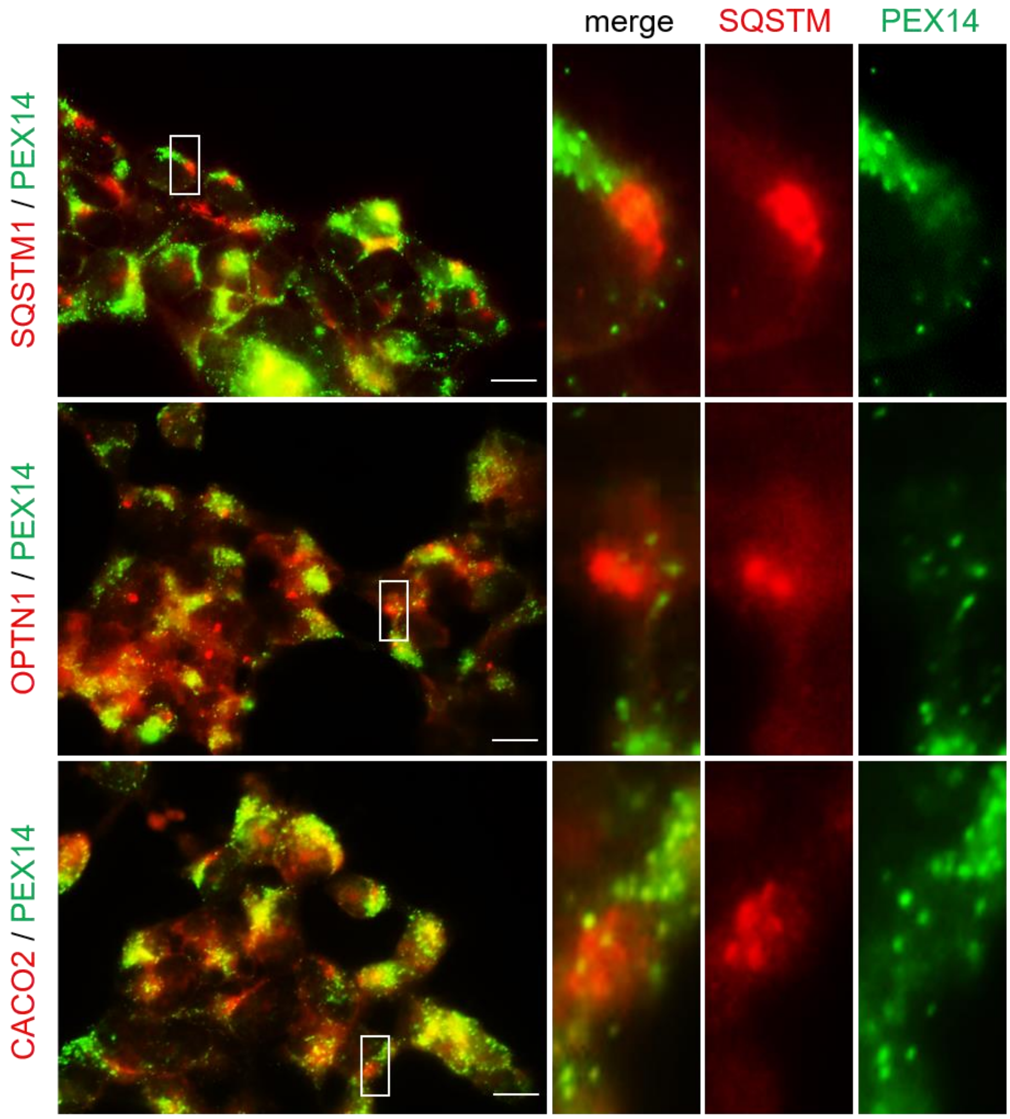

Figure S11. The specific autophagy receptors SQSTM1, OPTN, and CACO2 are not recruited to peroxisomes upon excessive production of po-H_2O_2. DD-DAO Flp-In T-REx 293 cells were cultured as described in the legend to Figure 3. Next, the cells were incubated in DBPS containing 10 mM 3-amino-1,2,4-triazole and 10 mM L- or D-Ala. After 60 min, the cells were fixed and processed for immunofluorescence microscopy using a mouse antiserum against PEX14 and rabbit antisera against SQSTM1, OPTN, or CACO2 in combination with goat anti-mouse and anti-rabbit secondary antibodies conjugated to Alexa Fluor 488 and Texas Red, respectively. The panels shown to the right of each image are magnified views of the boxed areas. Scale bars, 10 μm.

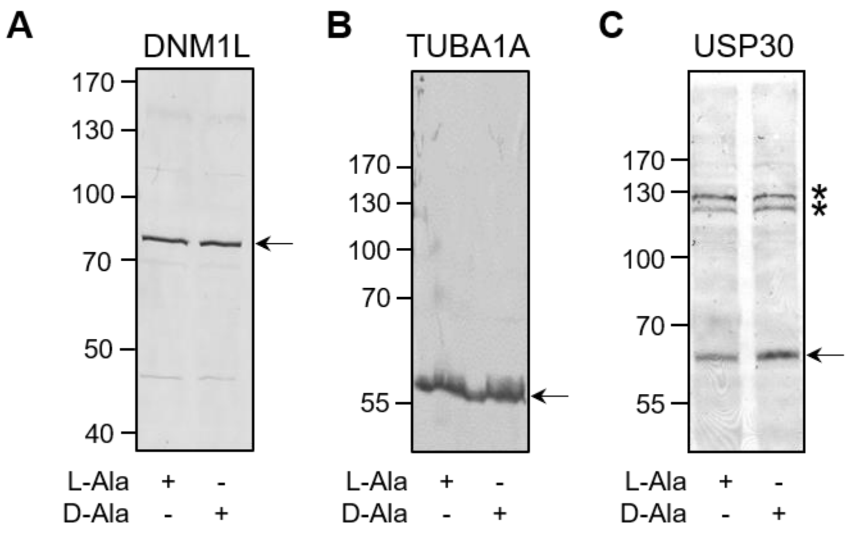

Figure S12. The ubiquitously expressed housekeeping protein TUBA1A, the ubiquitin-specific protease USP30, and the dynamin-like protein DNM1L do not undergo disulfide bond formation upon po-H_2O_2 production. DD-DAO Flp-In T-REx 293 cells were cultured as described in the legend to Figure 3. Next, the cells were incubated in DBPS containing 10 mM 3-amino-1,2,4-triazole and 10 mM L- or D-Ala. After 60 min, the free thiol groups were blocked with N-ethylmaleimide. Thereafter, the cells were processed for SDS-PAGE under non-reducing conditions and subsequently subjected to immunoblot analysis with antibodies specific for (**A**) DNM1L, (**B**) tubulin alpha 1a (TUBA1A), or (**C**) USP30. The migration points of relevant molecular mass markers (expressed in kDa) are shown on the left. The arrows and asterisks mark, respectively, the specific and non-specific protein bands.
